# West Mexico Berries Modulate α-Amylase, α-Glucosidase and Pancreatic Lipase Using In Vitro and In Silico Approaches

**DOI:** 10.3390/ph15091081

**Published:** 2022-08-30

**Authors:** Carmen Alejandrina Virgen-Carrillo, Elia Herminia Valdés Miramontes, David Fonseca Hernández, Diego A. Luna-Vital, Luis Mojica

**Affiliations:** 1 Behavioral Feeding and Nutrition Research Institute, University Center from the South, University of Guadalajara, Ciudad Guzman 49000, Jalisco, Mexico; 2 Food Tecnology, Centro de Investigación y Asistencia en Tecnología y Diseño del Estado de Jalisco, Guadalajara 44270, Jalisco, Mexico; 3 Tecnologico de Monterrey, The Institute for Obesity Research, Ave. Eugenio Garza Sada 2501, Monterrey 64849, Nuevo Leon, Mexico

**Keywords:** raspberry, blueberry, blackberry, freeze-drying, antioxidant potential, α-amylase, α-glucosidase

## Abstract

The objective was to evaluate the antioxidant and biological potential of eight freeze-dried berry varieties of southern Jalisco using in silico and in vitro approaches. Fourteen tentative phenolic compounds were identified in berries by ESI-QToF, including anthocyanins, phenolic acids, flavanols and flavonols. In silico assays of phytochemicals in the berry inhibiting enzymes related to obesity and diabetes showed predicted binding energy interactions (ranging from −5.4 to −9.3 kcal/mol). Among the cultivars, antioxidant potential for DPPH IC_50_ ranged from 1.27 to 3.40 mg/mL, ABTS IC_50_ from 2.26 to 7.32 mg/mL and nitric oxide (NO) inhibition IC_50_ from 4.26 to 11.07 mg/mL. The potential to inhibit α-amylase IC_50_ ranged from 4.02 to 7.66 mg/mL, α-glucosidase IC_50_ from 0.27 to 4.09 mg/mL, lipase IC_50_ from 1.30 to 4.82 mg/mL and DPP-IV IC_50_ from 1.36 to 3.31 mg/mL. Blackberry cultivars from the southern Jalisco region showed outstanding biological potential compared to other evaluated berries and could be used in the formulation of functional foods in the prevention of noncommunicable diseases.

## 1. Introduction

Mexican berries represent an important product in the national agricultural sector. Mexico ranks first place in blackberry production, second in raspberry and sixth in blueberry, worldwide [1]. Mexico has been ranked as the second exporter of these fruits worldwide [2]. Jalisco state (located in west Mexico) is the principal producer of blueberry and raspberry, and the second producer of blackberry in the country [1]. The global demand for berries is increasing and it is estimated that this demand will be duplicated by 2030. The growing demand for berries is mainly due to their health benefits. Extensive evidence has linked berries’ phytochemicals with the attenuation of chronic metabolic diseases [3].

Although multiple efforts have been applied by government agencies worldwide, cost-effective alternatives for the prevention and treatment of noncommunicable diseases (NCDs) such as diabetes and obesity are needed. Multiple studies have demonstrated that functional foods and nutraceuticals are excellent options in the development of new alternative treatments for these diseases. Bioactive compounds derived from food may influence health beyond nutrition [4]. Berry polyphenols exert anti-hyperglycemic, lipid-lowering and anti-inflammatory effects [5]. In the treatment of hyperglycemia, polyphenols could act through insulin-dependent and insulin-independent mechanisms. The insulin-dependent mechanism involves the enhancement of β-cell function and insulin sensitivity of peripheral tissue. Otherwise, the insulin-independent mechanism is related to the inhibition of digestive enzymes α-amylase and α-glucosidase which act by degrading polysaccharides [6]. Another mechanism to reduce glucose absorption is the inhibition of glucose transporters SGLT1 and GLUT2, and changes in energy metabolism via AMP-activated protein kinase that increases expression of GLUT4 and subsequently glucose uptake [5,6]. Another mechanism for reducing plasma glucose concentrations is the inhibition of the dipeptidyl peptidase IV (DPP-IV) enzyme, which acts by inactivating the glucose-like peptide 1 (GLP-1) and gastric inhibitory polypeptide (GIP). Incretin hormones are related to the insulin release from pancreatic β-cells [7,8]. Oxidative stress is related to hyperglycemia and obesity, and induces the overproduction of free radicals such as reactive oxygen species (ROS) and nitric oxide (NO) radicals, which generates pancreatic and kidney tissue damage. Berry secondary metabolites have been shown to inhibit ROS generation, protein carbonylation and nitration, lipid peroxidation and oxidative DNA damage [9].

Available literature evidencing bioactivity supports the use of berries in the formulation of functional foods for the prevention of non-communicable diseases.

Despite a large amount of evidence of the biological potential of the berry phenolic compounds against metabolic alterations, their concentration and biological potential are influenced by berry species, variety, growing conditions, cultivar, genetic factors, processing, transformation, and storage conditions [5,6]. In this sense, there is scarce information related to Mexican berries. The objective of this research was to evaluate the antioxidant and the biological potential of eight varieties of Mexican berries from southern Jalisco using in silico and in vitro approaches.

## 2. Results and Discussion

### 2.1. Total Polyphenols, Anthocyanins, Flavonoids, and Tannins

Table 1 shows the concentration of phenolic compounds identified in the extracts. Among cultivars, the highest concentration of total polyphenols was observed in the two varieties of blackberry with a mean of 10.46 mg GAE/g DW. Blueberry varieties had the lowest concentration (4.57–5.62 mg GAE/g DW), with no differences among them (*p >* 0.05). The variety Ras3 had a similar concentration (5.89 mg GAE/g DW) to blueberry varieties, but the lowest concentration compared to the other raspberry varieties. The phenolic composition in several berries has shown similarities with our findings; blackberries have the highest amount of phenols, followed by raspberries and blueberries [10,11,12,13]. Sariburun et al. [13] reported a total phenol content of four blackberry cultivars from 2279.8 ± 12.5 mg GAE/100 g FW (fresh weight) to 2786.8 ± 21.9 mg GAE/100 g FW; and the content of five raspberry cultivars from 1040.95 ± 15.91 mg GAE/100 g FW to 2062.27 ± 4.13 mg GAE/100 g FW. In Korean berries, Kim [11] reported a total phenolic composition of 1307.33 ± 9.24 mg GAE/100 g for blackberry, 936.67 ± 2.08 mg GAE/100 g for blueberry and 489.67 ± 2.52 mg GAE/100 g for raspberry. In Romanian berries, blueberries have obtained a higher value of polyphenols with 678 mg GAE/100 g FW, followed by blackberries (about 440 mg GAE/100 g FW) and raspberries with approximately 410 mg GAE/100 g FW [14].

For anthocyanin concentration, raspberry varieties showed the lowest concentration among cultivars (0.41–1.47 mg C3GE/g DW). Blueberry varieties showed the highest concentration (*p ˂* 0.05) among cultivars and similar concentration with blackberry varieties. The highest concentration was observed in Blu1 variety (3.61 mg C3GE/g DW). The highest concentration of anthocyanins has been reported in blackberries, followed by blueberries and raspberries [10,12,13]; however, in this work, one of the blueberry varieties showed the highest concentration among cultivars (4.98 ± 0.25 mg C3GE/g DW), while the other blueberry and blackberry varieties showed similarities, and raspberries had a significantly lower concentration (0.65 to 2.24 mg C3GE/g DW). Between four blackberry cultivars, total anthocyanins have been reported from 41.3 ± 0.3 mg C3GE/100 g FW to 87.1 ± 1 mg C3GE/100 g FW; and from five raspberry cultivars, from 12.4 ± 0.3 mg C3GE/100 g FW to 69.5 ± 0.5 mg C3GE/100 g FW [13].

For flavonoid concentration, the Blu3 variety presented the highest value (5.74 mg RUE/g DW), and no differences were observed between Blu2 and Blu1 (*p* > 0.05). Similar concentrations were obtained in Bla1 and Bla2 varieties with a mean of 4.28 mg RUE/g DW. The lowest concentration was observed in the Ras1 variety. This value was almost half of the maximum concentration obtained in the Blu3 variety. Similar to our findings, Sariburun et al. [13] found a higher amount of total flavonoids in blackberry varieties (29.07 ± 0.49 mg/100 g of (+)-catechin equivalents (CTE) to 82.21 ± 1.34 mg CTE/100 g FW), than in raspberries (15.41 ± 0.12 mg CTE/100 g FW to 41.08 ± 0.92 mg CTE/100 g FW).

For tannin concentrations, heterogeneous ranges were obtained among cultivars. The highest concentration of tannins was observed in blackberries varieties (112.05 ± 49.9 and 119.64 ± 68.7 mg CAE/g DW), the Ras2 raspberry variety (112.53 ± 9.4 mg CAE/g DW) and the Blu1 blueberry variety (107.24 ± 39.22 mg CAE/g DW), while the rest of the varieties showed lower concentrations. In contrast, Diaconease et al. [14] found the highest tannin values in blueberries (160 mg/100 g), followed by raspberries (120 mg/100 g) and blackberries (around 75 mg/100 g).

### 2.2. Antioxidant Potential (AP) from Different Berry Cultivars

All the berry varieties studied showed an important AP with the three different methods (ABTS, DPPH, NO). The results were expressed as IC_50_ values that, at the lowest levels, indicate the highest free radical-scavenging activity of the samples.

**Figure 1:**
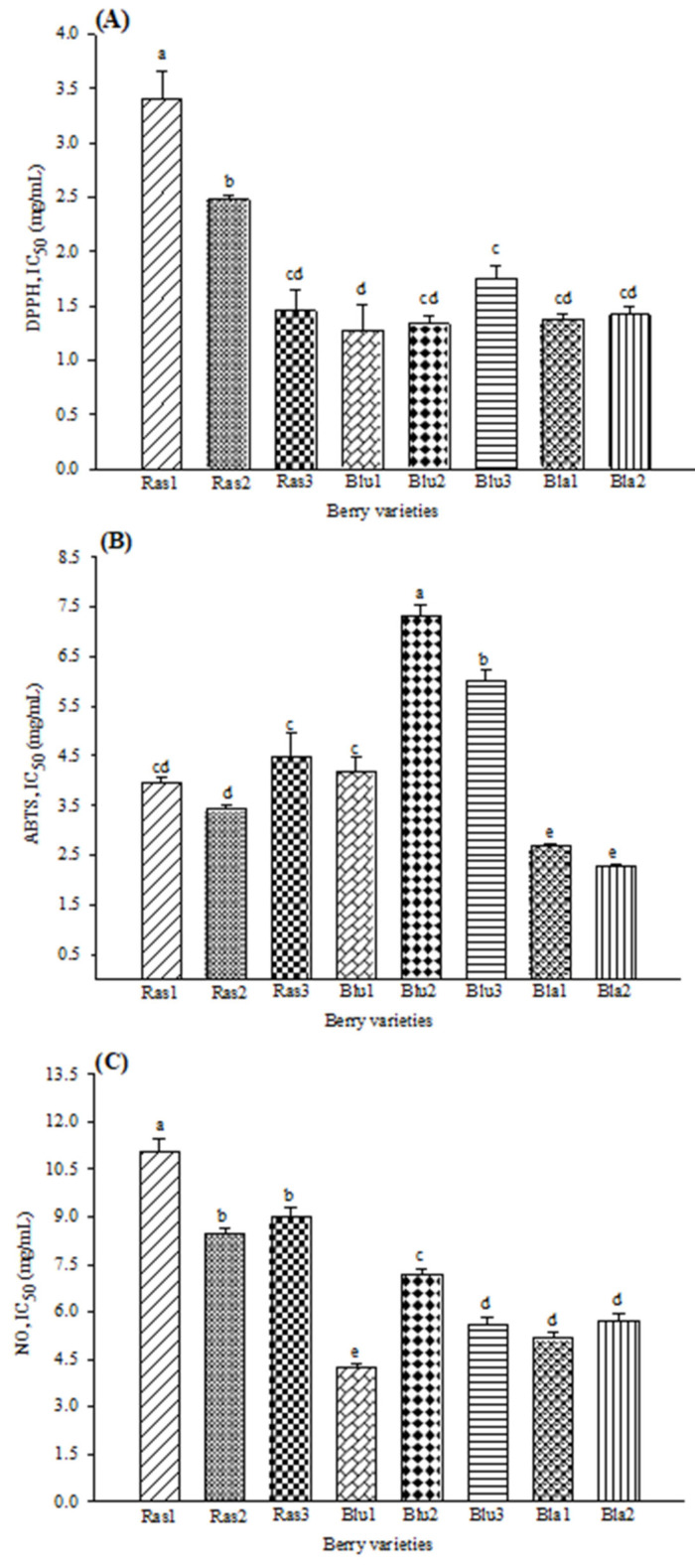
Antioxidant activity in eight varieties of Mexican berries, showing the scavenging of (**A**) DPPH radical, (**B**) ABTS radical and (**C**) nitric oxide. Data represent the mean ± SD from three independent replicates. Different letters (a–e) indicate significant difference (*p* < 0.05) according to Tukey’s test. DPPH: 2,2-diphenyl-1-picrylhydrazyl; ABTS: 2,2′-azino-bis 3-ethylbenzothiazoline-6-sulphonic acid; NO: nitric oxide. IC50 values (mg/mL).

In general, AP results (Figure 1) show that Bla1 and Bla2 varieties have the best radical-scavenging activity. Moreover, the lowest activity for DPPH and NO was for raspberry varieties, while for ABTS, blueberry varieties showed the lowest inhibition capacity. For the DPPH assay, Figure 1A shows that the highest inhibition was in the Blu1 variety (IC_50_ 1.27 mg/mL), with no differences with Bla1 and Bla2 varieties (IC_50_ 1.37 and 1.41 mg/mL, respectively). For ABTS inhibition (Figure 1B), the highest activity observed was in the Bla1 (IC_50_ 2.69 mg/mL) and Bla2 (IC_50_ 2.26 mg/mL) blackberry varieties. As shown in Figure 1B, the lowest activity was obtained in the Blu2 variety (IC_50_ 7.32 mg/mL). Figure 1C shows that the Blu1 variety had the highest inhibition activity at 4.26 mg/mL concentration for the NO inhibition assay. Moreover, the lowest inhibition was observed in the Ras1 raspberry variety (IC_50_ 11.07 mg/mL).

The antioxidant potential of berries has been widely reported. Diverse methods such as ORAC, FRAP, DPPH and ABTS have been used to evaluate the antioxidant capacity of blackberry, blueberry, and raspberry [11,12,15]. Similar findings were obtained by Sariburun et al. [13] using the DPPH and ABTS methods; while, for NO, the highest potential was observed in the Blu1 blueberry variety and blackberries varieties. Free radical scavenging promotes the reduction of diabetes mellitus (DM) complications associated with oxidative stress, such as β cell apoptosis and insulin resistance.

### 2.3. Biological Potential of Berries Cultivars

**Figure 2:**
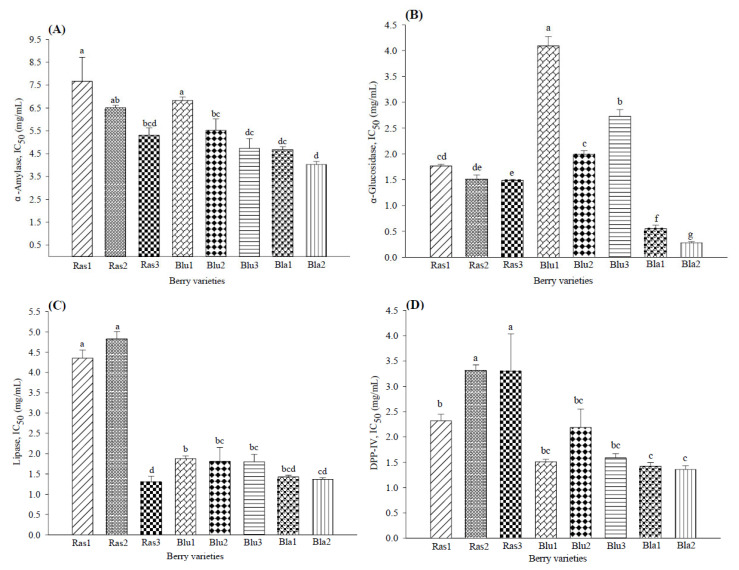
Biological activity of eight varieties of Mexican berries in biochemical assays inhibiting (**A**) α-amylase, (**B**) α-glucosidase, (**C**) lipase and (**D**) dipeptidyl peptidase IV. Data represent the mean ± SD from three independent replicates. Different letters (a–g) indicate significant difference (*p* < 0.05) according to Tukey’s test. DPP-IV: dipeptidyl peptidase IV.

Modulation of glucose metabolism markers by phenolic compounds present in berries has been related to the inhibition of α-glucosidase and DDP-IV activity by anthocyanins and α-amylase activity by ellagitannins [8,10]. In this experiment, all berry varieties inhibited the in vitro activity of these enzymes. Figure 2 shows that the lowest concentration required to inhibit α-glucosidase, α-amylase and DPP-IV activity was observed in blackberries (IC_50_ of 4.02 ± 0.12, 0.27 ± 0.02 and 1.36 ± 0.07 mg/mL, respectively), followed by some blueberry varieties (Figure 2A–D). Among the blackberry varieties, Bla2 showed the highest bioactivity presenting the lowest IC_50_ values for all the assays. The highest potential activity shown in blackberries has also been reported by Fan et al. [7] (from 0.07 to 0.22 IC_50_ µM) and Johnson et al. [10] (from 5.5 to 18.2 IC_50_ µM).

The IC_50_ for pancreatic lipase inhibition (Figure 2C) indicated the highest activity by blackberries (1.36 to 1.42 mg/mL), followed by the Ras3 raspberry variety (IC_50_ 1.30 ± 0.14 mg/mL) and blueberry varieties (IC_50_ 1.80 to 1.87 mg/mL), while the lowest activity was shown by Ras1 and Ras2 (IC_50_ 4.35 and 4.82 mg/mL). Similarly, McDougall et al. [16] reported that a tannin-rich raspberry extract effectively inhibited pancreatic lipase activity in vitro, while blueberry caused a slight inhibition.

### 2.4. Tentative Identification of Phenolic Compounds

As seen in Table 2, fourteen phenolic compounds were tentatively identified in analyzed samples, including the anthocyanins: malvidin-3-glucoside, delphinidin 3-glucoside, cyanidin 3-glucoside, petunidin-3-*O*-beta-glucoside, pelargonidin-3-glucoside and peonidin-3-*O*-glucoside; the flavonol quercetin-3-d-galactoside; the flavanols: catechin and epicatechin; and the phenolic acids: ellagic acid, *p*-coumaric acid, gallic acid, caffeic acid and salicylic acid.

Quercetin-3-D-galactoside and cyanidin 3-glucoside were identified in all berry varieties; malvidin-3-glucoside and delphinidin 3-glucoside were identified in blueberry and blackberry varieties; petunidin-3-*O*-beta-glucoside and peonidin-3-*O*-glucoside were found in raspberries and blueberries; pelargonidin-3-glucoside was found in raspberries and blackberries; (-) epicatechin, ellagic acid and *p*-coumaric were identified in raspberry varieties; gallic acid and caffeic acid were identified in blueberry varieties; while catechin and salicylic acid were only identified in blackberries.

Similar to our findings, in Romanian, Finnish and Turkish raspberry varieties, quercetin-3-d-galactoside [15], cyanidin 3-glucoside [12,13,17], petunidin-3-*O*-beta-glucoside [12], epicatechin [17], ellagic acid [15,17] and *p*-coumaric acid [17] have been identified. Some other compounds have been found in raspberry cultivars such as ellagic acid-pentoside, ellagic acid-acetyl-xyloside, quercetin-galactosyl-rhamnoside, quercetin-glucosyl-rutinoside, quercetin-3-glucoside, quercetin-3-glucuronide, cyanidin 3-sophoroside, cyanidin 3-rutinoside, cyanidin 3-glucosylrutinoside, pelargonidin-3-glucoside and pelargonidin-3-rutinoside [12,13,15,17].

In Romanian blueberries, Diaconeasa et al. [15] identified some of the compounds we found, such as quercetin-3-d-galactoside and caffeic acid; likewise, Marhuenda et al. [12] identified malvidin-3-glucoside, delphinidin 3-glucoside, cyanidin 3-glucoside, petunidin-3-*O*-beta-glucoside and peonidin-3-*O*-glucoside. In addition, these authors also found quercetin-rutinoside, quercetin-glucoside and chlorogenic acid in these fruits. Meanwhile, none of them identified gallic acid. In Romanian, Brazilian and Turkish blackberry varieties, phenolic compounds have been identified similar to our results, such as quercetin-3-d-galactoside [15], catechin [18], delphinidin 3-glucoside [12], salicylic acid [18], cyanidin 3-glucoside [12,13] and pelargonidin-3-glucoside [13]. Additional compounds have been identified in blackberry cultivars such as myricetin-galactoside, myricetin-arabinoside, quercetin-rutinoside, quercetin-glucoside, ellagic acid, gallic acid, gallocatechin, syringic acid, cyanidin 3-rutinoside, cyanidin 3-xyloside, cyanidin, malvidin, pelargonidin and delphinidin [12,13,15,18]. Meanwhile, malvidin-3-glucoside was also identified in blackberry varieties.

### 2.5. Molecular Docking of Berry Phytochemicals on Enzymes Related to Obesity and Diabetes

According to UPLC-ESI-QToF-MS/MS analysis, the extracts may contain diverse phenolic compounds that are present in other berry varieties. However, it is important to use pure standards to confirm the presence of phenolic acids and specific flavonoids.

**Figure 3:**
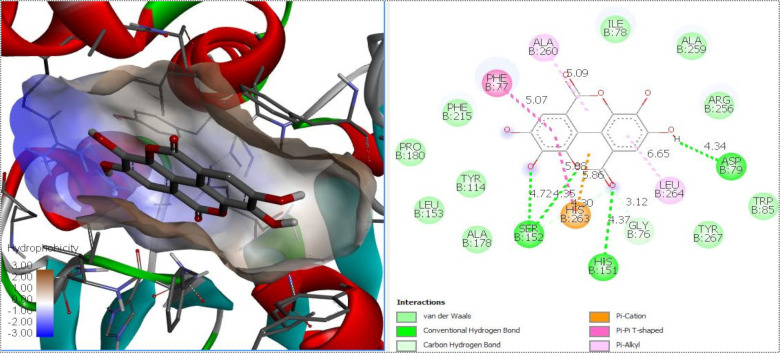
Molecular docking diagram of ellagic acid in the catalytic site of pancreatic lipase.

In computational docking analysis, the theoretical free energy values showed the potential of the berries’ bioactive compounds to interact with the target enzyme. Table 3 shows the minimum estimated free energy of polyphenols identified in berry extracts with α-glucosidase, α-amylase, DPP-IV and lipase. Figure 3 shows an example of ellagic acid in the catalytic site of pancreatic lipase. Interaction with lipase presented negative affinity values, from −6.1 kcal/mol (gallic acid) to −9.3 kcal/mol (ellagic acid). For DPP-IV, the binding energies were −5.4 kcal/mol (salicylic acid) to −7.4 kcal/mol (ellagic acid). The theoretical binding energy for α-glucosidase ranged from −5.4 kcal/mol (malvidin 3-*O*-glucoside and salicylic acid) to −7.7 kcal/mol (ellagic acid), while for α-amylase, the energy ranged from −5.9 kcal/mol (salicylic acid) to −8.9 kcal/mol (catechin). For positive controls, orlistat presented a free energy value of −6.2 kcal/mol for lipase; sitagliptin had a free energy value of −6.7 kcal/mol for DPP-IV; and acarbose presented a free energy value of −6.6 and −7.2 kcal/mol for α-glucosidase and α-amylase, respectively. Among the interaction of polyphenols with pancreatic lipase, DPP-IV, α-glucosidase and α-amylase showed similar binding energies compared to positive controls (orlistat, sitagliptin, acarbose). However, most of the berry polyphenols showed a higher inhibition potential compared to drugs.

For lipase, *p*-coumaric acid, gallic acid, salicylic acid and caffeic acid presented a similar affinity to inhibit the enzyme, compared to the positive control; conversely, the rest of the identified compounds showed greater inhibition potential compared to Orlistat. The strongest theoretical activity was shown by ellagic acid (−9.3 kcal/mol). The main theoretical interactions between ellagic acid and the catalytic site of lipase were through hydrogen and Van Der Waals bonds, as well as cation-π interactions. In another case, molecular docking analysis showed that caffeic acid, *p*-coumaric acid and quercetin were able to inhibit the activity of lipase [19]. In our biochemical study results, the highest inhibition potential was shown by phenolic compounds, ellagic acid, (-) epicatechin and catechin, which were exclusively identified in raspberry and blackberry varieties.

Johnson et al. [10] reported that anthocyanins identified from blueberries and blackberries had a strong potential to inhibit DPP-IV enzyme, similarly to the control diprotin A; conversely, Fan et al. [7] demonstrated that flavonoids from blueberry–blackberry wine could inhibit DPP-IV enzyme. In our case, most of the compounds inhibit the enzyme with similar values compared to sitagliptin; however, cyanidin 3-glucoside, quercetin 3-d-glucoside and ellagic acid showed a stronger potential.

Zhang et al. [20] demonstrated that anthocyanidins identified in blueberries could be efficiently docked into the catalytic site of α-glucosidase. In our study, (-)-epicatechin (−7.3 kcal/mol), catechin (−7.5 kcal/mol) and ellagic acid (−7.7 kcal/mol) showed a greater potential to inhibit α-glucosidase enzyme than control acarbose (−6.6 kcal/mol); conversely, some of the rest had binding energies similar to the control.

Most of the compounds showed higher activity than acarbose (−7.2 kcal/mol) to inhibit α-amylase; however, catechin (−8.9 kcal/mol) stood out for presenting the highest potential. Sui et al. [21] demonstrated that the anthocyanins peonidin-3-glucoside (−7.46 kcal/mol), cyanidin-3,5-glucoside (−7.15 kcal/mol), cyanidin-3-glucoside (−6.75 kcal/mol) and cyanidin-3-rutinoside (−6.53 kcal/mol) showed competitive inhibition against porcine pancreatic α-amylase due to its binding to the active site.

Blackberry varieties showed the greatest potential to inhibit α-glucosidase and α-amylase by biochemical analysis. This result was similar to the in silico analysis in which the catechin, only identified in blackberries, demonstrated a potential inhibition greater than acarbose. As can be seen from these results, some compounds showed higher potential than the positive controls to inhibit the enzymes.

## 3. Materials and Methods

### 3.1. Chemicals

Folin and Ciocalteu’s phenol reagent, 2-Aminoethyl diphenylborinate 97%, ABTS (2,2′-Azino-bis-(3-ethylbenzothiazoline-6-sulphonic acid), DPPH: 2,2-Diphenyl-1-picrylhydrazyl, Trolox^®^: (±)-6-Hydroxy-2,5,7,8-tetramethylchromane-2-carboxylic acid, MOPS: 3-(N-Morpholino) propanesulfonic acid, 4-Morpholinepropanesulfonic acid (≥99.5%), Orlistat: tetrahydrolipstatin (≥98%), DMSO: Dimethyl sulfoxide, α-Amylase from porcine pancreas (EC3.2.1.1), α-Glucosidase from *Saccharomyces cerevisiae* (EC3.2.1.20), lipase from porcine pancreas (EC3.1.1.3), Dipeptidyl Peptidase IV human (EC 3.4.14.5), quercetin-3-d-galactoside (≥98%), delphinidin 3-*O*-glucoside (≥98%), malvidin 3-*O*-glucoside (≥98%) and cyanidin 3-*O*-glucoside (≥98%), acarbose (≥95%), 3,5-Dinitrosalicylic acid (≥98%), *p*-Nitrophenyl-α-d-glucopyranoside (≥99%), catechin (99%), rutin (94%), gallic acid (97.5%), vanillin (99%), sodium nitroprusside dihyrate (≥99%), sitagliptin, tributyrin, sodium taurodeoxycholate hydrate, 4-Nitrophenol, *tert*-butanol and Griess reagent, were purchased from Sigma Aldrich (St. Louis, MO, USA). DPP-IV GLO^®^ protease assay kit G8351 was purchased from Promega (Madison, WI, USA). All chemicals were analytical grade.

### 3.2. Sample Collection

Three varieties (Ras1, Ras2, Ras3) of raspberry (*Rubus idaeus*), three (Blu1, Blu2, Blu3) of blueberry (*Vaccinium* spp.) and two (Bla1, Bla2) of blackberry *(Rubus ulmifolius*) were obtained by cultivar zones in southern Jalisco during one production cycle (December 2018–January 2019). Fresh fruits were stored at −20 °C until the lyophilization process. After, frozen fruits were separated individually and lyophilized using a Harvest Right™ freeze dryer. The freeze-dried samples were stored in a vacuum package until required for analysis.

### 3.3. Polyphenol-Rich Extracts Preparation

Lyophilized berries samples were grounded using an IKA^®^ A11 analytical mill, then were sieved in 40 US-mech (standard testing sieve, Advantech a W.S. Tyler® Company, Mentor, OH, USA) and stored in a plastic bag. For every independent replication, an acidified ethanol extract (85:15 ethanol:HCl) was obtained in a 1:10 (solid:solvent) ratio of the powdered sample of each fruit. Sonication at room temperature (3 times × 15 min each) in an Ultrasonic Cleaner SB-5200DTN (Ningbo Scientz^®^ Biotechnology Co., Ltd., Tech Park, Ningbo, Zhejiang, China) was used to assist the extraction, followed by centrifugation for 10 min at 10,000 rpm. The supernatant was collected in an Eppendorf^®^ microtube and stored at −20 °C until analysis.

### 3.4. Analysis of Extracts Composition

#### 3.4.1. Total Phenolic Compounds

Total phenolic compounds were measured using the Folin–Ciocalteu method adapted to a microassay using gallic acid (GA) as standard [22]. Samples were diluted to a factor of 1:10 and 1:20 with deionized water. Then, 50 µL microliters of the samples, GA standard (40–100 μg/mL) or blank (deionized water) were placed in a 96-well flat-bottom plate and then 50 µL of 1 N Folin–Ciocalteu’s phenol reagent were added. After 5 min, 100 µL of 20% Na_2_CO_3_ were added, and the mixture was incubated for 10 min. The absorbance was read at 690 nm in a Tecan Infinite^®^ M200 PRO (Tecan Group, Ltd., Männedorf, Switzerland). Final concentrations were expressed as mg of gallic acid equivalents (GAE) per g of DW (dry weight) (y = 0.0094x − 0.0521, R^2^ = 0.9975).

#### 3.4.2. Total Anthocyanins

Total monomeric anthocyanins were determined by the pH differential method [23]. Samples were diluted to a factor of 1:10 and 1:50 using two buffers (potassium chloride buffer, pH 1.0, 0.25 M; sodium acetate buffer, pH 4.5, 0.4 M). Then, 200 µL of diluted solutions at each pH were transferred to a 96-well flat-bottom plate, and the absorbance was read at 520 and 700 nm using a Tecan Infinite^®^ M200 PRO (Tecan Group, Ltd., Männedorf, Switzerland). Anthocyanin concentration was calculated as cyanidin-3-*O*-glucoside (C3G) equivalents per L as below; where: A = [pH 1.0 (A_520_−A_700_) − pH 4.5 (A_520_−A_700_)]; MW (molecular weight) = 449.2 for C3G; DF = dilution factor; 1000 = conversion factor from grams to milligrams; PL = constant path length 0.6217 cm; *ε* = 26,900 L/moL-cm is the molar extinction coefficient for C3G. Final concentrations were expressed as mg C3G equivalents per g of DW.

$$Total\;monomeric\;anthocyanins\;\left(\mathrm{mg}/L\right)=\frac{A\;\times \;\mathrm{MW}\;C3G\;\times \;\mathrm{DF}\;\times 1000}{\varepsilon \times \;\mathrm{PL}}$$

#### 3.4.3. Flavonoids

Total flavonoid concentration was quantified using the method reported by Mojica et al. [22] with some modifications. Briefly, 50 μL of each sample, previously diluted to a factor of 1:20 with deionized water or rutin standard (1–50 μg/mL), were placed in a 96-well flat-bottom plate, followed by the addition of 180 μL of methanol 99.8% and 20 μL 1% 2-aminoethyldiphenyl borate/methanol solution. For the blank, 230 μL of methanol 99.8% and 20 μL 1% 2-aminoethyldiphenyl borate/methanol solution were added. The absorbance was read at 404 nm using a Tecan Infinite^®^ M200 PRO (Tecan Group, Ltd., Männedorf, Switzerland). Final concentrations were expressed as mg rutin equivalents (RUE) per g of DW, (y = 0.0027x − 0.0004, R^2^ = 0.9994).

#### 3.4.4. Tannins

For total tannins concentration, catechin (CAE) was used as standard. The method was done as reported by Mojica et al. [22]. Samples of extract were diluted to a factor of 1:20 and 1:40 with deionized water. In a 96-well flat-bottom plate, 50 µL of these diluted samples, CAE standard (0.1–0.8 mg/mL) or blank (50 µL of methanol 99.8% and 200 μL of 4% acidified methanol) were added, followed by the addition of 200 μL of 8% acidified methanol and 1% vanillin methanol solution to a factor of 1:1. The absorbance was read at 500 nm using a Tecan Infinite^®^ M200 PRO (Tecan Group, Ltd., Männedorf, Switzerland). Final concentrations were expressed as mg catechin equivalents (CAE) per g of DW (y = 0.2279x + 0.0167, R^2^ = 0.997).

### 3.5. Antioxidant Potential

#### 3.5.1. DPPH Assay

DPPH (1,1-diphenyl-2-picrylhydrazyl) assay was carried out according to the method reported by Luna-Vital et al. [24], with some modifications. Samples were diluted to a factor of 1:20 to 1:100 with phosphate-buffered saline (PBS) 0.01 M, pH 7.4. In a 96-well flat-bottom plate, 20 µL of the diluted samples, Trolox standard (0.01–0.275 mM) or blank (PBS) were loaded, followed by the addition of 180 μL of 0.06 mM DPPH in 80% methanol, and then incubated in the dark for 30 min. After incubation, the absorbance was read at 517 nm using a Tecan Infinite^®^ M200 PRO (Tecan Group, Ltd., Männedorf, Switzerland). Trolox was used for the calibration curve (y = −0.6969x + 0.2129, R^2^ = 0.99). Results were presented as IC_50_ values (mg/mL) and were determined from the free radical scavenging activity (%).

#### 3.5.2. ABTS

ABTS (2,2-azinobis-3-ethylbenzothiazoline-6-sulphonic acid) assay was performed according to the method reported by Luna-Vital et al. [24], with some modifications. Samples were diluted to a factor of 1:5 to 1:70 deionized water. In a 96-well flat-bottom plate, 20 µL of the diluted samples, Trolox standard (0.01–0.275 mM) or blank (deionized water) were added, followed by the addition of 180 μL of 7 mM ABTS^+^ radical solution. The absorbance was read at 734 nm using a Tecan Infinite^®^ M200 PRO (Tecan Group, Ltd., Männedorf, Switzerland). Trolox was used as a standard for the calibration curve (y = −1.184x + 0.603, R^2^= 0.99). Results were presented as IC_50_ values (mg/mL) and were determined from the free radical scavenging activity (%).

#### 3.5.3. Nitric Oxide (NO) Radical Scavenging Assay

Scavenging of NO was determined as previously reported by Oseguera-Toledo et al. [25], with some modifications. Sodium nitroprusside (SNP) was used as the NO donor. Samples were diluted to a factor of 1:5 to 1:40 with phosphate-buffered saline 0.01 M, pH 7.4. In a 96-well flat-bottom plate, either 50 µL of the diluted sample or blank (PBS) were incubated with SNP solution at 27 °C. After 120 min, 50 µL of the incubated solution was mixed with 50 µL of Griess reagent and incubated again for 10 min. After a time, absorbance was immediately read at 550 nm using a Tecan Infinite^®^ M200 PRO (Tecan Group, Ltd., Männedorf, Switzerland). Results were presented as IC_50_ values mg/mL and were determined from the free radical scavenging activity (%).

### 3.6. Biological Potential

#### 3.6.1. α-Amylase Inhibition Biochemical Assay

For the α-amylase assay, the method was used as reported by Mojica et al. [23], with some modifications. Samples were diluted to a factor of 1:10 to 1:40 with PBS 0.01 M, pH 7.4. In microtubes, 50 µL of each diluted sample, 1 mM acarbose (positive control), or PBS, 0.02 M, pH 6.9 (negative control) were added to 50 µL of 13 U/mL α-amylase solution (PBS 0.02 M, pH 6.9), and incubated at 37 °C for 10 min. Afterward, 50 µL of 1% soluble starch solution (previously dissolved in PBS 0.02 M, pH 6.9, and boiled for 10 min) was added to each tube and incubated at 37 °C for another 10 min. Finally, 1 mL of dinitro salicylic acid reagent (DNS) was added, and the tubes were placed in a 100 °C water bath for 5 min. The mixture was diluted with 1 mL of distilled water. In a 96-well plate, 200 µL of each mixture was added, and absorbance was read at 540 nm. The same procedure was carried out in microtubes, where 50 µL of PBS 0.02 M, pH 6.9 solution were added instead of α-amylase solution. The results obtained by these three independent experiments were considered to calculate the inhibition percentage with the following equation, where: A = the subtraction of the absorbance obtained in the sample with α-amylase enzyme solution and the absorbance obtained in the sample with PBS solution; B = mean of the absorbance obtained in the negative control PBS with α-amylase enzyme. Results were presented as IC_50_.

$$\%Inhibition=\left(\frac{B-A}{A}\right)$$

#### 3.6.2. α-Glucosidase Inhibition Biochemical Assay

The assay was based on the method reported by Mojica et al. [23], with some modifications. Each sample of acidified ethanol extract was evaporated to remove ethanol with a Thermo Scientific™ Savant, SpeedVac™ Concentrator (Thermo Fisher Scientific Inc., Waltham, MA, USA). Deionized water was added to obtain the initial volume before evaporation. For the α-glucosidase assay, samples were diluted to a factor of 1:40 to 1:700 with PBS (0.01 M, pH 7.4). In a 96-well flat-bottom plate, either 50 µL of the diluted sample, 1 mM acarbose (positive control), or PBS, 0.1 M, pH 6.9 (negative control) were added to 100 µL of 1 U/mL α-glucosidase solution (PBS, 0.1 M, pH 6.9) and incubated at 37 °C for 10 min. Later, 50 µL of 5 mM p-nitrophenyl-α-d-glucopyranoside solution (PBS 0.1 M, pH 6.9) was added to each well and incubated at 37 °C for 5 min. Absorbance was read at 405 nm. Results were presented as IC_50_ values (mg/mL).

#### 3.6.3. Dipeptidyl Peptidase IV (DPP-IV) Inhibition Biochemical Assay

DPP-IV inhibition was measured as reported previously by Mojica et al. [23] using the DPP-IVGLO^®^ Protease Assay (G8351, Promega, Madison, WI, USA). Samples were diluted to a factor of 1:10 to 1:100 with deionized water. In a white-walled 96-well plate, 40 µL of these diluted samples, sitagliptin (positive control, 50–500 nm) or buffer pH 8.0: 100 mM TRIS, 200 mM NaCl, 1 mM EDTA (negative control) were added, followed by the addition of 10 μL of DPP-IV human enzyme (10 ng/mL) and 50 μL of DPP-IVGLO^®^ reagent. The blank contained only 50 µL of buffer pH 8.0 and 50 µL of DPP-IVGLO^®^ reagent. Luminescence was measured after mixing and incubating for 30 min at 27 °C using a SpectraMax^®^ i3 Multi-Mode Microplate Reader (Molecular Devices^®^, LLC; San Jose, CA, USA). Results were presented as IC_50_ values (mg/mL).

#### 3.6.4. Lipase Inhibition Biochemical Assay

Lipase inhibition activity was determined by using PHIBLA (pH Indicator-Based Lipase Assay) as reported by Camacho-Ruiz et al. [26] with some modifications. Each sample of the extract was diluted to a factor of 1:10 to 1:100 with PBS (0.01 M, pH 7.4). To measure indirectly the release of free fatty acids by lipase-catalyzed hydrolysis of short-chain tributyrin substrate TG (4:0), 100 mM and 4-nitrophenol (pH indicator) were dissolved in *tert*-butanol. A substrate emulsion (5 mM) was obtained by vigorous mixing on a vortex of 8:1:1 proportion of buffer solution, TG (4:0) and 4-nitrophenol. Buffer solution included 2.5 mM MOPS, 0.5 mM NaTDC, 150 mM NaCl and 6 mM CaCl_2_. In a 96-well flat-bottom plate, either 10 µL of the diluted sample, or Orlistat (positive control), or buffer (negative control) were added to 10 µL of lipase enzyme solution and incubated at 37 °C for 60 min in a microplate shaker. After incubation, 100 μL of substrate emulsion (prepared no more than 3 min early) was quickly added using an eight-channel pipette. Immediately, the plate was placed in a microtiter plate scanning spectrophotometer (x-Mark^TM^, Bio-rad Laboratories, Inc. Hercules, CA, USA) and shaken for 5 s before each reading. The decrease in absorbance at a wavelength corresponding to the λmax of the pH indicator was recorded at 410 nm every 30 s at 37 °C. Results were presented as IC_50_ values (mg/mL).

#### 3.6.5. Tentative Identification of Phenolic Compounds by UPLC-ESI-QToF-MS/MS

Phenolic compound tentative identification was made according to Fonseca-Hernández et al. [27]. A chromatographic method was performed on a Waters Acquity UPLC H-Class system (Milford, MA, USA). Chromatographic separation was done at 40 °C with a flow rate of 0.3 mL/min on an Acquity UPLC BEH C18 column (2.1 × 50 mm, 1.7 µm). The mobile phase consisted of two eluent solvents: (A) acetonitrile with 0.3% formic acid and (B) Mili-Q water 0.3%. The gradient elution was as follows: 90% A at 0–1 min, 90–30% A at 1–11 min, 30–90% A at 11–12 min, 90% at 12–15 min. The injection volume was set at 8 µL.

Mass spectrometry (MS) analysis was performed on a Water Xevo G2-XS QToF quadrupole time-of-flight mass spectrometer, with an electrospray ionization (ESI) interface (Milford, MA, USA). The MS acquisition was operated in negative ion mode, with a mass range of *m*/*z* 50 to 800. The parameters were: capillary voltage: 2.5 kV, cone voltage: 40 kV, source temperature: 100 °C, desolvation temperature: 250 °C, collision energy: 6.0 eV. MassLynx 4.1 MS software (Milford, MA, USA) was used to process data.

#### 3.6.6. Molecular Docking Analysis

Molecular interactions of polyphenols identified in the extracts and the catalytic site of the enzymes were evaluated by molecular docking. Crystal structures of human lipase (1LPB), dipeptidyl peptidase IV (1RWQ), α-glucosidase (3AJ7) and α-amylase (2QV4) were obtained from the Protein Data Bank (https://www.rcsb.org/, accessed on 4 August 2021). Positive controls of orlistat, sitagliptin and acarbose were obtained from PubChem (https://pubchem.ncbi.nlm.nih.gov/, accessed on 4 August 2021). Docking analyses were performed with Autodock Tools software and AutoDock Vina (version v.1.2.0., The Scripps Research Institute, La Jolla, CA, USA). [28] The lowest binding energy was selected to be visualized in the Discovery Studio Visualizer version v21.1.0.20298 (BIOVIA, San Diego, CA, USA).

### 3.7. Statistical Analysis

Statistical analysis was evaluated considering at least three independent replications. The data were analyzed using the Statistical Package for the Social Sciences version 24 (IBM^®^ SPSS Statistics, Chicago, ILL, USA). One-way analysis of variance (ANOVA) with Tukey’s post hoc analyses were used to determine differences among fruits and varieties. An α level of *p* < 0.05 was considered statistically significant. Data are expressed as means ± standard deviation. The IC_50_ and correlation among parameters measured were calculated using GraphPad Prism (Version 8.0.2; GraphPad Software, Inc.; San Diego, CA, USA). The graphs were made with the SigmaPlot 10.0 software for Microsoft Windows.

## 4. Conclusions

The differences between studies results in the concentration of compounds, their antioxidant capacity and their biological activity are associated to the method of extraction, the solvent type, the variety, maturity of the berry, storage, cultivation conditions and environmental conditions, among others [13,15,18]. Despite this, it can be inferred that our results highlight the characteristics of blackberries among the reviewed studies and in the results of this work, regardless of the variety, geographical area of cultivation, extraction method and some other conditions mentioned previously.

In conclusion, this work presents important new information related to berries grown in the region of Jalisco, Mexico. These compounds could act and interact synergistically to enhance the antioxidant, hypoglycemic and lipid-lowering effects observed in in vitro tests. The evaluated blackberry varieties could be highlighted as the fruits that showed the best results both for the in vitro and in silico assays. These results could be useful in the development of nutraceutical products that can be evaluated in clinical trials and may impact the development of economically sustainable alternative treatments in the prevention and management of metabolic diseases.

## Figures and Tables

**Table 1:** Phenolic compound concentration in eight varieties of Mexican berries.

Fruit Sample	Variety	TPC (mg GAE/g DW)	Anthocyanins (mg C3GE/g DW)	Flavonoids (mg RUE/g DW)	Tannins (mg CAE/g DW)
Raspberry	Ras1	8.05 ± 0.32 ^bc^	0.45 ± 0.04 ^e^	2.62 ± 0.11 ^d^	37.94 ± 2.32 ^a^
	Ras2	9.66 ± 0.30 ^b^	1.55 ± 0.08 ^d^	3.45 ± 0.86 ^dc^	112.53 ± 9.4 ^a^
	Ras3	6.55 ± 0.35 ^cd^	0.65 ± 0.04 ^e^	2.39 ± 0.20 ^d^	49.47 ± 1.41 ^a^
Blueberry	Blu1	5.79 ± 0.17 ^d^	3.44 ± 0.17 ^a^	4.68 ± 0.21 ^ab^	107.24 ± 39.22 ^a^
	Blu2	5.08 ± 0.66 ^d^	2.25 ± 0.27 ^bc^	4.60 ± 0.22 ^ab^	84.24 ± 36 ^a^
	Blu3	5.91 ± 0.22 ^d^	2.80 ± 0.24 ^b^	5.37 ± 0.37 ^a^	110.42 ± 56.7 ^a^
Blackberry	Bla1	11.94 ± 1.19 ^a^	2.29 ± 0.22 ^bc^	4.41 ± 0.16 ^abc^	112.05 ± 49.9 ^a^
	Bla2	11.32 ± 0.63 ^a^	2.15 ± 0.31 ^c^	4.15 ± 0.30 ^bc^	119.64 ± 68.7 ^a^

Data represent the mean ± SD from at least three independent replicates. Means within a column followed by different letters (^a–e^) were significantly different (*p* < 0.05) according to Tukey’s test. TPC: total phenolic compounds; GAE: gallic acid equivalents; C3GE: cyaniding 3 glucoside equivalents; RUE: rutin equivalents; CAE: catechin equivalents; DW: dry weight.

**Table 2:** Tentative identification of metabolite compounds in extracts of eight varieties of Mexican berries.

Tentative Identification	Elemental Formula	Variety Sample	*m*/*z* Experimental	*m*/*z* Theoretical	tR
Quercetin-3-d-Galactoside *	C_21_H_20_O_12_	Ras1	463.1829	463.1211	4.86
		Ras2	463.1829	463.1211	4.97
		Ras3	463.1829	463.1211	4.70
		Blu1	463.1741	463.1211	4.79
		Blu2	463.1741	463.1211	4.79
		Blu3	463.1741	463.1211	4.80
		Bla1	463.1829	463.1211	4.75
		Bla2	463.1829	463.1211	4.69
Malvidin-3-Glucoside *	C_23_H_25_O_12_	Ras1	-	-	-
		Ras2	-	-	-
		Ras3	-	-	-
		Blu1	491.1996	491.119	5.94
		Blu2	491.2086	491.119	3.91
		Blu3	491.2086	491.119	5.95
		Bla1	491.2813	491.119	6.24
		Bla2	491.2813	491.119	6.28
Delphinidin 3-Glucoside *	C_21_H_20_O_12_	Ras1	-	-	-
		Ras2	-	-	-
		Ras3	-	-	-
		Blu1	301.1333	301.0349	6.58
		Blu2	301.1333	301.0349	6.56
		Blu3	301.1262	301.0349	6.68
		Bla1	301.0977	301.0349	4.80
		Bla2	301.0977	301.0349	4.69
Cyanidin 3-Glucoside *	C_21_H_21_O_11_^+^	Ras1	447.1505	447.1242	4.96
		Ras2	447.1505	447.1242	4.97
		Ras3	447.1505	447.1242	5.29
		Blu1	447.1764	447.1242	5.33
		Blu2	447.1764	447.1242	5.33
		Blu3	447.1764	447.1242	5.35
		Bla1	447.1851	447.1242	2.98
		Bla2	447.1851	447.1242	3.11
Petunidin-3-*O*-Beta-Glucoside	C_22_H_23_O_12_	Ras1	477.16	477.1033	4.86
		Ras2	477.16	477.1033	4.97
		Ras3	477.16	477.1033	4.70
		Blu1	477.1868	477.1033	5.33
		Blu2	477.1957	477.1033	5.33
		Blu3	477.1764	477.1033	5.35
		Bla1	-	-	-
		Bla2	-	-	-
Pelargonidin-3-Glucoside	C_21_H_21_O_10_	Ras1	431.3153	431.0978	12.33
		Ras2	431.3068	431.0978	12.33
		Ras3	431.3068	431.0978	12.45
		Blu1	-	-	-
		Blu2	-	-	-
		Blu3	-	-	-
		Bla1	431.2898	431.0978	4.07
		Bla2	431.2898	431.0978	4.09
Peonidin-3-*O*-Glucoside	C_22_H_23_O_11_	Ras1	461.2635	461.1084	4.20
		Ras2	461.2722	461.1084	5.18
		Ras3	461.2546	461.1084	4.21
		Blu1	461.2019	461.1084	3.91
		Blu2	461.2019	461.1084	3.99
		Blu3	461.2019	461.1084	3.91
		Bla1	-	-	-
		Bla2	-	-	-
(-) Epicatechin	C_15_H_14_O_6_	Ras1	289.1659	289.0712	3.79
		Ras2	289.1659	289.0712	3.84
		Ras3	289.1659	289.0712	3.79
		Blu1	-	-	-
		Blu2	-	-	-
		Blu3	-	-	-
		Bla1	-	-	-
		Bla2	-	-	-
Ellagic acid	C_14_H_6_O_8_	Ras1	301.0977	301.0358	4.72
		Ras2	301.0906	301.0358	4.70
		Ras3	301.0977	301.0358	4.70
		Blu1	-	-	-
		Blu2	-	-	-
		Blu3	-	-	-
		Bla1	-	-	-
		Bla2	-	-	-
*p*-Coumaric acid	C_9_H_8_O_3_	Ras1	-	-	-
		Ras2	163.1315	163.0395	4.42
		Ras3	163.1315	163.0395	4.21
		Blu1	-	-	-
		Blu2	-	-	-
		Blu3	-	-	-
		Bla1	-	-	-
		Bla2	-	-	-
Gallic acid	C_7_H_6_O_5_	Blu1	169.0798	169.0606	9.03
		Blu2	169.0798	169.0606	5.33
		Blu3	169.0824	169.0606	5.35
Caffeic acid	C_15_H_10_O_4_	Blu1	179.0695	179.0345	4.16
		Blu2	179.0641	179.0345	0.52
		Blu3	179.1408	179.0345	1.67
Catechin	C_15_H_14_O_6_	Bla1	289.1659	289.1219	3.79
		Bla2	289.1729	289.1219	3.96
Salicylic acid	C_7_H_6_O_3_	Bla1	137.1133	137.0249	5.72
		Bla2	137.1133	137.0249	5.70

tR: retention time, * symbol indicates the theoretical mass-to-charge ratio (m/z) obtained with pure standards, while theoretical *m*/*z* without the symbol was obtained from the MassBank of North America (MoNA) (https://mona.fiehnlab.ucdavis.edu/, accessed on 7 July 2021).

**Table 3:** Molecular docking predictions.

Compound	Lipase (kcal/mol)	Dipeptidyl Peptidase IV (kcal/mol)	α-Glucosidase (kcal/mol)	α-Amylase (kcal/mol)
Quercetin 3-d-Glucoside	−8.7	−7.1	−5.7	−8.4
Malvidin 3-*O*-Glucoside	−7.7	−6.7	−5.4	−7.8
Delphinidin 3-*O*-Glucoside	−8.5	−6.9	−6.4	−8.0
Cyanidin 3-Glucoside	−8.6	−7.0	−6.4	−8.3
Petunidin 3-*O*-Glucoside	−8.5	−6.9	−6.4	−8.0
Pelargonidin 3-Glucoside	−8.7	−6.8	−5.5	−8.0
Peonidin 3-*O*-Glucoside	−8.5	−6.3	−6.2	−8.5
(-) Epicatechin	−9.2	−6.9	−7.3	−8.6
Ellagic acid	−9.3	−7.4	−7.7	−8.6
*p*-Coumaric acid	−6.2	−6.4	−5.6	−6.4
Gallic acid	−6.1	−6.4	−5.5	−6.4
Caffeic acid	−6.7	−6.7		−6.7
Catechin	−9.2	−6.6	−7.5	−8.9
Salicylic acid	−6.2	−5.4	−5.4	−5.9
Orlistat	−6.2	-	-	-
Sitagliptin	-	−6.7	-	-
Acarbose	-	-	−6.6	−7.2

Theoretical binding affinity (kcal/mol) of polyphenols identified in berry extracts. “-“ indicates that the compound was not tested as an inhibitor.

## Data Availability

Data is contained within the article.
